# Sp7 Action in the Skeleton: Its Mode of Action, Functions, and Relevance to Skeletal Diseases

**DOI:** 10.3390/ijms23105647

**Published:** 2022-05-18

**Authors:** Hironori Hojo, Shinsuke Ohba

**Affiliations:** 1 Center for Disease Biology and Integrative Medicine, Graduate School of Medicine, The University of Tokyo, Tokyo 113-0033, Japan; hojo@g.ecc.u-tokyo.ac.jp; 2 Department of Cell Biology, Institute of Biomedical Sciences, Nagasaki University, Nagasaki 852-8588, Japan; 3 Department of Oral Anatomy and Developmental Biology, Osaka University Graduate School of Dentistry, Osaka 565-0871, Japan

**Keywords:** Sp7, osteoblasts, skeleton

## Abstract

Osteoblast differentiation is a tightly regulated process in which key transcription factors (TFs) and their target genes constitute gene regulatory networks (GRNs) under the control of osteogenic signaling pathways. Among these TFs, Sp7 works as an osteoblast determinant critical for osteoblast differentiation. Following the identification of Sp7 and a large number of its functional studies, recent genome-scale analyses have made a major contribution to the identification of a “non-canonical” mode of Sp7 action as well as “canonical” ones. The analyses have not only confirmed known Sp7 targets but have also uncovered its additional targets and upstream factors. In addition, biochemical analyses have demonstrated that Sp7 actions are regulated by chemical modifications and protein–protein interaction with other transcriptional regulators. Sp7 is also involved in chondrocyte differentiation and osteocyte biology as well as postnatal bone metabolism. The critical role of SP7 in the skeleton is supported by its relevance to human skeletal diseases. This review aims to overview the Sp7 actions in skeletal development and maintenance, particularly focusing on recent advances in our understanding of how Sp7 functions in the skeleton under physiological and pathological conditions.

## 1. Introduction

Mammalian skeletons are derived from the ectoderm and mesoderm [1]. The neuroectoderm-originated neural crest exclusively develops into craniofacial skeletal elements, including facial bones, the frontal bone, the squamous part of the temporal bone, and the anterior part of the sphenoid bone [2,3]. The remainder of the skeleton is formed by the two mesoderm derivatives. The paraxial mesoderm gives rise to the sclerotome via somites, forming the parietal bone, the occipital bone, the petrous part of the temporal bone, the posterior part of the sphenoid bone, the vertebral column, and the ribs [2,3]. The lateral plate mesoderm develops into the sternum and the appendicular skeleton [2,3].

Two distinct modes of ossification underlie skeletal formation from these origins: intramembranous ossification and endochondral ossification. The cranial vault, most of the facial bones, and the body of the clavicle are generated by the former, whereas the cranial base, the mandibular condyle, the vertebral column, the sternum, the ribs, the end of the clavicle, and the appendicular skeleton are generated by the latter [3,4]. In both processes, mesenchymal cells are first condensed at regions where skeletons are supposed to develop. In intramembranous ossification, cells in the condensed mesenchyme directly undergo the osteoblast differentiation process, which generates bone-forming osteoblasts [3,4]. In endochondral ossification, chondrogenesis precedes osteogenesis: mesenchyme-derived osteo-chondroprogenitors differentiate into chondrocytes, which form cartilage models. Following the formation of bone-forming osteoblasts, which also originated from the osteo-chondroprogenitors, the cartilage is replaced by mineralized bone tissues.

Osteoblast differentiation is a tightly regulated process in which key transcription factors (TFs) and their target genes constitute gene regulatory networks (GRNs) under the control of osteogenic signaling pathways. Those TFs and signaling pathways include runt Runx2 (related transcription factor 2), Sp7/Osterix, Dlx (distal-less homeobox), Msx (msh homeobox), ATF4 (activating transcription factor 4), Wnt/β-catenin signaling, Hedgehog (Hh) signaling, bone morphogenetic protein (BMP) signaling, transforming growth factor-β (TGF-β) signaling, fibroblast growth factor (FGF) signaling, and Notch signaling (extensively reviewed in [5]). Among these factors, Runx2 and Sp7/Osterix (Sp7 hereinafter) are known as master regulators for osteoblast differentiation; deletion of either of them led to a complete lack of osteoblasts in both endochondral and intramembranous bones [6,7]. In osteoblast differentiation, skeletal progenitors (including osteo-chondroprogenitors) are initially specified into *Runx2*-positive osteoblast precursors and committed to the osteoblast lineage; the *Runx2*-positive osteoblast precursors then become *Runx2*–*Sp7*-double-positive osteoblast precursors [8]. The *Runx2*–*Sp7*-double-positive precursors differentiate into osteoblasts and osteocytes, contributing to the formation and maintenance of bone tissues and bone marrows [9].

For more than a decade after its discovery in 2002, Sp7 had been thought to be an osteoblast determinant, which induced transcription of osteoblastic genes by directly binding to their regulatory regions on the genome. However, a next-generation sequencer (NGS)-based genome-scale search for Sp7-associated regions indicated that a “non-canonical” mode of DNA binding might be the preferred action of Sp7 on the osteoblast genome; emerging evidence further supports the presence of such a “non-canonical” mode, even in other Sp family members, and its relevance to human diseases. In addition, recent studies suggest the potential roles of Sp7 in the terminal differentiation of chondrocytes and osteocyte biology as well as osteoblastogenesis. In this review, we, therefore, aim to summarize and discuss the actions of Sp7 in skeletal tissues, particularly focusing on recent advances in the understanding of its modes of action on the genome, its broader biological functions in skeletal cells, its genomic targets, the regulation of its expression and functions, and its clinical relevance.

## 2. Sp7: A Master Regulator of Osteoblast Differentiation

Sp7 was identified by screening for genes that were differentially expressed in BMP-2-treated C2C12 cells compared to untreated ones [7]. The mouse *Sp7* gene is located at chromosome 15, whereas human *SP7* is found at chromosome 12q13.13 [7]. The *Sp7* gene is composed of three exons and two introns. Rapid amplification of 5′ complementary DNA ends (5′ RACE) identified three alternatively spliced variants of *Sp7*. The organization of the *Sp7* gene appears to be conserved between mice and humans [10,11]. In human *SP7*, the type I isoform is generated by removal of both introns and exon 2; the type II isoform by removal of introns 1 and 2; and the type III isoform by removal of only intron 2. The expected protein products are identical between type II and type III because of the absence of an initiation codon in exon 1 and the identical coding sequence between the two isoforms, while the protein from the type I transcript lacks the first 18 N-terminal amino acids [10]. Although these Sp7 isoforms are expressed in different cell types [10], functional differences among the isoforms, i.e., differences in transactivation, have not been clarified yet.

The gene was initially called Osterix (Osx). Its current official gene symbol is Sp7; it contains three C2H2-type zinc fingers—which have high homology with those in Sp family transcription factors Sp1, Sp3, and Sp4—at its C terminus. Based on the following observations, as well as the presence of C2H2-type zinc fingers, Sp7 was thought to have typical properties of TFs. First, Sp7 bound to the GC-box in an electrophoretic mobility-shift assay (EMSA), as Sp1 did. Second, Sp7 had a proline-rich transactivation domain at its N terminus. Third, its subcellular localization was restricted to the nucleus.

In the developing mouse skeleton, *Sp7* transcripts first appear in chondrocytes and the surrounding perichondrium of endochondral bones and in the condensed mesenchyme of intramembranous bones at embryonic day (E) 13.5. Strong *Sp7* expression is detected in the osteoblast lineage located in primary spongiosa and the perichondrium/bone collars at E15.5 and later. The expression is also detectable in prehypertrophic chondrocytes in the growth plate. At the postnatal stages, Sp7 is expressed in osteoblasts on the surface of trabecular bones, the endosteum, and the periosteum and in osteocytes in the bone matrix [7,12].

In the initial report that identified Sp7, the critical role of Sp7 in osteoblast differentiation was demonstrated by means of a mouse genetic study [7]. *Sp7*-null mice died immediately after birth due to breathing difficulty, although the mice with heterozygous deletion of *Sp7* appeared normal and fertile. *Sp7*-null embryos completely lacked bone formation in both endochondral and intramembranous bones, although their cartilage development remained normal. *Sp7*-null mice failed to express osteoblast-related genes, including *Sparc* (secreted protein acidic and cystein rich; also known as *osteonectin*), *Spp1* (secreted phosphoprotein 1, also known as *osteopontin*), *Ibsp* (integrin-binding sialoprotein, also known as *bone sialoprotein*), and *Bglap* (bone gla protein, also known as *osteocalcin*) and showed severely reduced expression of *Col1a1*, the main component of bone matrix, in their skeletal elements. Given that *Runx2* is expressed in *Sp7*-null skeletal elements, it seems likely that osteoblast differentiation is arrested at the *Runx2*-positive stage in *Sp7*-null mutants. The absence of *Sp7* expression in *Runx2*-null mice further suggests that Sp7 acts genetically downstream of Runx2 [7]. Lastly, *Sp7*-null cells that failed to differentiate into osteoblasts ectopically expressed chondrocyte regulator and cartilage matrix genes, including *Sox5* (SRY-box transcription factor 5), *Sox9* (SRY-box transcription factor 9), *Ihh* (Indian hedgehog), and *Col2a1*, in both endochondral and intramembranous bones. Thus, the phenotypes of *Sp7*-null mice indicate that Sp7 acts as a critical determinant for bone-forming osteoblasts in skeletal development.

Several studies also support the postnatal roles of Sp7 in bone homeostasis. Inducible but ubiquitous deletion of *Sp7* at postnatal stages caused a reduction of new bone formation; there were few mature osteoblasts, and *Col1a1* expression was severely decreased in the long bones of the mutants [13]. The mutants also showed a decreased number of osteocytes with few dendrites. The expression of the osteocyte-related genes *Dmp1* (dentin matrix protein 1), *Phex* (phosphate regulating endopeptidase homolog X-linked), and *Sost* (sclerostin) was reduced in the mutants [13]. Osteoblast-specific deletion of *Sp7* using a 2.3-kb *Col1a1-Cre* driver line rescued the perinatal lethality of *Sp7*-null mice, and the newborn mutants appeared normal. However, they showed osteopenic phenotypes at growing stages, probably due to a delay of osteoblast maturation [14]. Inducible deletion using a 2.3-kb *Col1a1-CreERT2* driver line basically yielded phenotypes similar to those of the above mutants [15]. These results suggest that Sp7 positively regulates osteoblast differentiation and subsequent osteocyte formation in postnatal bones. Given that overexpression of *Sp7* in osteoblasts using a transgenic approach resulted in osteopenia with suppression of osteoblast differentiation [16], an appropriate dosage of *Sp7* may be required for the proper execution of the osteoblast program in adults.

## 3. The Mode of Sp7 Action: How Does It Contribute to the Transcription of Osteoblastic Genes as an Osteoblast Determinant?

Until fairly recently, conventional mechanistic studies on the actions of TFs had focused on local gene regulation, i.e., how the TFs regulate transcription of certain sets of target genes, and this approach provided a good model for understanding their modes of action. However, it remained unclear whether the models were relevant to the overall actions of the TFs on the genome and to their cell-type distinct functions. Next-generation sequencer (NGS)-based genome-scale studies have recently addressed these questions, uncovering how TFs are associated with the genome, how they potentially interact with other proteins over the genome, and how the actions potentially elicit biological outcomes.

A chromatin immunoprecipitation-sequencing (ChIP-seq) study unveiled an Sp7-DNA association profile in mouse calvaria-derived primary osteoblasts and mouse pre-osteoblastic cell line MC3T3-E1, showing several key features of Sp7 actions on the osteoblast genome [17]. First, Sp7-associated regions, i.e., Sp7 ChIP-seq peak regions, were found to be enriched in distal regulatory elements (>5 kb from gene bodies), suggesting that Sp7 is involved in the long-range interaction between enhancers and promoters. Second, the sequences of Sp7-associated regions were well-conserved among vertebrate species. Third, genes that were related to skeletal development and expressed in skeletal tissues were significantly enriched in putative target gene sets of the Sp7-associated regions, which were predicted by their proximity; correlation analysis with the transcriptome data obtained from *GFP*-positive osteoblasts of neonatal *Sp7*-*GFP* mouse calvarias [8] further revealed that Sp7 ChIP-seq signals were most enriched around osteoblast-specific genes. These data suggest that Sp7-mediated long-range interactions commonly underlie the transcription of osteoblastic genes among vertebrates.

The last but most important feature of the actions of Sp7 was found by de novo motif analysis on Sp7-associated regions. The analysis revealed enrichment of an AT-rich sequence under the Sp7-associated regions. This was unexpected because Sp7 belongs to the Sp family; Sp family members bind to the GC-box consensus motif via their zinc finger domains [18]. The mode of action was confirmed by ChIP-seq analysis for Sp1, Sp2, and Sp5 [19,20,21] and high throughput screening of protein-DNA bindings for Sp1, Sp3, and Sp4 [22,23]. Sp7 was also shown to bind to the GC box in vitro, as Sp1 did [7].

In a comparative analysis of Sp1 and Sp7 ChIP-seq in the pre-osteoblastic cell line MC3T3-E1, the AT-rich motif was again enriched in Sp7-associated regions, whereas the GC-box was enriched in Sp1-associated ones [17]. The Sp7 binding affinity to the GC-box was much less than that of Sp1 and, in most cases, below the detection limit in biochemical analyses [17]. Sp7 was likely to lose the GC-box preference due to amino acid differences within the zinc finger domain since Sp7 acquired a binding affinity to the GC-box by three amino acid substitutions in the Sp7 zinc fingers (α-helical domain) to their Sp1 counterparts [17].

How is Sp7 associated with the AT-rich motifs on the osteoblast genome? No direct binding of Sp7 to the AT-rich motifs was detected in EMSA. The AT-rich motif resembled a homeodomain-responsive element, which raised the possibility that Sp7 is indirectly bound to the motif through homeodomain-containing TFs. Several lines of evidence supported the conclusion that homeodomain-containing Dlx factors act with Sp7. Dlx factors have been implicated in osteoblast differentiation [24,25,26]. Indeed, a ChIP-seq study in MC3T3-E1 demonstrated that almost 80% of Sp7-associated regions overlapped with Dlx5-associated regions [17]. Sp7 and Dlx5 physically interacted, and the Sp7–Dlx5 complex bound to the AT-rich motif in EMSA, whereas Sp7 alone did not [17]. The functional significance of the Sp7–Dlx5 interaction in osteoblasts was confirmed; *Dlx* knock-down attenuated Sp7 engagements with the osteoblast enhancers on the genome, leading to suppression of the Sp7 target gene expression [17].

**Figure 1:**
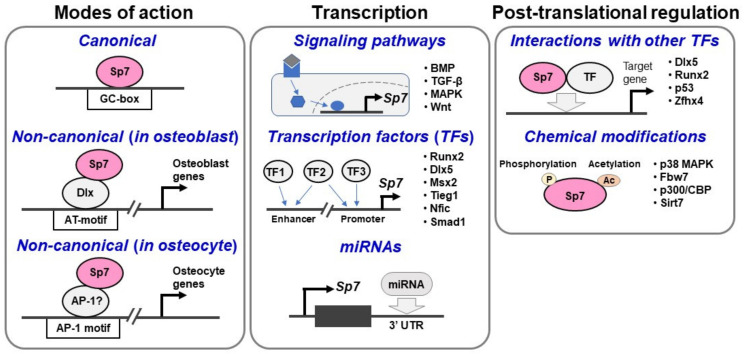
Current understanding of Sp7 actions and regulations. Modes of Sp7 action (**left**), transcriptional regulations of *Sp7* (**middle**), and post-translational regulations of Sp7 (**right**) activities are illustrated.

The above series of data suggests that Sp7 executes the osteoblast program via a “non-canonical” mode of action, in which it acts as a co-factor for Dlx rather than as a TF that directly binds to DNA. Other homeodomain-containing TFs, such as Msx1/2, Satb2 (special AT-rich sequence binding protein 2), and Alx4 (aristaless-like homeobox 4), expressed in osteoblasts [17] may bind to the AT-rich motif and interact with Sp7 on the osteoblast genome. In addition, the motifs of other key osteoblast TFs, such as Runx2 and Nfat (nuclear factor of activated T cells), were enriched in Sp7-associated regions [17]. These findings suggest that key regulatory inputs from multiple osteogenic signals are integrated into the Sp7-associated genomic regions in the osteoblast program (Figure 1).

Recent ChIP-seq studies suggest that the “non-canonical” mode of action is not specific to Sp7 but is present in other Sp family members as well. *Sp8* and *Sp6* are expressed in the limb ectoderm and play necessary roles for proximal–distal (PD) and dorsal–ventral (DV) patterning in limb development. Motif analysis on the Sp8 ChIP-seq in the mouse limb ectoderm showed that a GC-rich motif was the most over-represented one, and an AT-rich motif was the second-most over-represented. This suggests that Sp8 has a dual mode of action, direct DNA binding through the typical Sp consensus motif and indirect binding through homeodomain-containing TFs. Indeed, physical interactions were detected between Sp8 and Dlx5 and between Sp6 and Dlx5. However, the functional outcome of the interaction seems to be complex and likely depends on the availability of the interacting TFs [27]. Sp6 ChIP-seq analysis in developing mouse teeth similarly demonstrated that an AT-rich motif was enriched in the Sp6-bound genomic regions [28].

As mentioned earlier, amino acid variants in the α-helical domain distinguish Sp7 from other Sp family members, causing loss of the GC-box preference. Interestingly, a sequence comparison among vertebrate and non-vertebrate chordate species revealed that the closest non-boney vertebrates (e.g., lampreys), the cephalochordates (e.g., amphioxus), and the ascidians (e.g., tunicate) do not have Sp7-like zinc finger variants, whereas all boney vertebrates and cartilaginous fish that arose from a boney ancestor have Sp7 genes or a gene with Sp7-like zinc finger variants [17]. Therefore, Sp7 is likely to have appeared with the emergence of bone-forming osteoblasts, acting as an evolutionary switch in the cartilage-to-bone transition in the evolution of boney vertebrates.

## 4. Targets of Sp7 in Osteoblasts

Sp7 has been shown to upregulate transcription of various osteoblast-related genes, including *Fmod* (fibromodulin) [29], *Col1a1* [30,31], *Col1a2* [32], *Col5a1* [33], *Col5a3* [34], *Ibsp* [29,35], *Sost* [13,36], *Bglap* [37], *Zbtb16* [38], *Cx43* [39], *Vegf* (vascular endothelial growth factor) [40], *Mmp9* (matrix metalloproteinase 9) [41], *Mmp13* (matrix metalloproteinase 13) [42,43], *Zip1* [44], *Ucma* (upper zone of growth plate and cartilage matrix associated) [45], and *Enpp1* (pyrophosphatase/phosphodiesterase 1) [46] (extensively reviewed in [47]). This series of studies demonstrated that Sp7 bound to the typical GC-box (Sp1-binding sites) or CCAAT sequences around these genes (Figure 1).

The aforementioned Sp7 ChIP-seq study [17] confirmed the association of Sp7 with previously reported osteoblast enhancers around *Runx2* [48] and *Col1a1* [49]. It is worth noting that the Sp7 ChIP-seq confirms the previous findings and provides mechanistic support. One example is related to the work by Kawane et al. [48]. They found an association of multiple osteoblastic TFs, including Dlx5/6, Msx2, and Sp7, with the identified *Runx2* enhancer by ChIP. However, Dlx5 and Msx2, but not Sp7, are directly bound to the core sequence of the enhancer in EMSA. Since a physical interaction between Dlx5 and Sp7 was detected by GST pulldown, they proposed an enhanceosome model of the *Runx2* enhancer, in which Dlx5/6 is directly bound to the core-sequence, and Sp7 indirectly did so by forming a complex with Dlx5/6. The Sp7 ChIP-seq study at least partly confirmed the model and, more importantly, further expanded it to a genome scale (Figure 1).

The Sp7 ChIP-seq study also revealed a set of enhancers as novel genomic targets of Sp7. Site-directed mutations in AT-rich motifs within these targets suppressed their enhancer activities [17], suggesting that Sp7 acts on the elements through the AT-rich motif. In addition, one of the Sp7-bound enhancers located in *Notch2* intron 1 was confirmed as an osteoblast-specific enhancer; in transgenic reporter mouse analysis, its enhancer activity was found to be associated with Sp7 expression in the osteoblast lineage and was not detected in chondrocytes despite the expression of Sp7 in prehypertrophic chondrocytes [17]. Sp7 ChIP-seq peaks around *Notch2*, *Gli2*, *Fgfr2* (fibroblast growth factor receptor 2), and *Kremen1* further suggested cross-talk between Sp7 and osteogenic signaling pathways; their signaling activity is likely fine-tuned by Sp7 at the transcriptional level.

## 5. Roles of Sp7 in Chondrocytes and Osteocytes

Although global *Sp7*-null mouse embryos had no abnormality in cartilage, chondrocyte-specific deletion of *Sp7* using a *Col2a1-Cre* driver line resulted in impaired endochondral ossification with reduced expression of chondrocyte-related genes [50]. Another study using the *Col2a1-Cre* driver line showed that the calcification of cartilage matrix was delayed in the mutants [43]. Postnatal deletion of *Sp7* in chondrocytes using a *Col2a1-CreERT2* driver line leads to reduced trabecular bone mass in mice. This phenotype was accompanied by a delay of chondrocyte hypertrophy and cartilage-to-bone conversion [51]. Thus, these data suggest that Sp7 may have some impact on chondrocyte differentiation, particularly in the late stage of chondrocyte differentiation, and on the subsequent replacement of cartilage by bones during endochondral ossification. However, it remains to be elucidated how Sp7 is connected with GRNs in in vivo chondrocyte differentiation; genome-scale profiling of endogenous Sp7-DNA association in the chondrocyte lineage would be necessary to address this question.

Sp7 also has a crucial role in osteocyte biology [13,52], and a recent study provides insight into the molecular mechanisms underlying the Sp7-mediated functions [53]. *Sp7* deletion in mature osteoblasts using a *Dmp1-Cre* driver line led to severe defects in osteocyte dendrites in mice. RNA-seq in *Sp7*-deficient and *Sp7*-overexpressing Ocy454 cells, a mouse osteocyte-like cell line, revealed that Sp7-dependent genes were enriched in gene ontology (GO) terms linked to cell projection organization and neuronal development. Comparative analysis of Sp7 ChIP-seq between Ocy454 cells and primary osteoblasts revealed Ocy-specific Sp7-associated regions, which were connected with genes associated with axon guidance. These results suggest that the osteocyte network bears similarity to the network of intercellular connections between neurons in terms of both gene expression and morphology [54]. ChIP-seq analysis further provided insights into the mode of Sp7 action in osteocytes. Motif analysis showed that the Ocy-specific Sp7-associated regions had selective enrichment for the TGA(G/T)TCA motif bound by AP-1 family members. Therefore, Sp7 is likely to cooperate with distinct transcription factors to regulate enhancer activities in osteoblasts and osteocytes (Figure 1).

## 6. Transcription of Sp7

*Sp7* transcription is regulated by signaling pathways, TFs, and miRNAs, as recently reviewed in detail by Liu et al. [47]. With respect to the signaling-pathway-mediated regulation, in brief, BMP, TGF-β, mitogen-activated protein kinase (MAPK), and Wnt pathways are all involved in *Sp7* regulation (Figure 1). As for TFs, Runx2, Dlx5, Msx2, Tieg1, and Nfic are involved (Figure 1). Most of the molecules involved in these two types of regulation were examined by mechanistic analyses with *Sp7* promoter assays. Among these regulators, BMP signaling is particularly notable, given that Sp7 was originally identified as a gene upregulated in BMP-2-treated C2C12 cells. BMP signaling has been demonstrated to activate *Sp7* transcription in both a Runx2-dependent and a Runx2-independent manner [55]. In the Runx2-dependent mechanism, BMP signaling induces *Runx2*, which in turn activates *Sp7* transcription. In contrast, Smads are thought to regulate *Sp7* in the Runx2-independent mechanism. Recently, Smad1 enrichment was detected in the conserved region at 13-kb upstream of the *Sp7* transcription start site (TSS) by ChIP in E13.5 mouse limb bud cells treated with BMP-2 [56]. This may partly support the existence of Smad-mediated *Sp7* transcription.

In addition to these findings, we recently revealed that Runx2 controls *Sp7* transcription through a novel *Sp7* enhancer, which is located at approximately 11 kb distal to the *Sp7* transcription start site [57]. An analysis with a transgenic reporter mouse showed that the enhancer was activated in skeletal elements exclusively in osteoblasts but not prehypertrophic chondrocytes, suggesting that the genomic region acts as an osteoblast-specific *Sp7* enhancer. EMSA showed the direct binding of Runx2 on the Runx consensus motif in the enhancer; mutagenesis on the Runx consensus motif confirmed the requirement of the Runx2 binding site for the enhancer activity in vitro. Thus, a further analysis focusing on not only promoter regions but also enhancer regions will help to elucidate how *Sp7* is regulated in a cell type-specific manner.

MicroRNAs (miRNAs) and long non-coding RNAs (lncRNAs) were reported to participate in the Sp7 regulation (Figure 1). A number of miRNAs, including miR-27a and miR-96, were reported to have negative impacts on *Sp7* transcription through directly binding to 3′ untranslated region (UTR) of *Sp7* [47]; some miRNAs, such as miR-322 and miR-510, activate *Sp7* expression indirectly by regulating a negative factor of osteogenesis [47]. lncRNAs also have a variety of biological functions in the Sp7 regulation. lncRNA ODIR1 inhibited *Sp7* transcription by modifying histone marks, including H3K4me3 on the *Sp7* promoter [58], whereas lncRNA ob1 activated *Sp7* expression, possibly through inhibition of H3K27me3 on the promoter [59]. Overall, *Sp7* transcription is finely tuned through multiple mechanisms, although the whole picture of the regulation has not been revealed yet.

## 7. Post-Translational Regulation of Sp7 Activities

The activities of Sp7 are regulated post-translationally by the chemical modification of Sp7 and its interactions with other transcriptional regulators (Figure 1). p38 MAPK was shown to phosphorylate Sp7 at Ser-73 and Ser-77 [29]. The functional significance of the phosphorylation event was examined in terms of the Sp7 binding to the GC-box [29]. A mutant Sp7, in which the two p38 targets were mutated, resulted in decreased recruitment of RNA polymerase II, p300, and the chromatin remodeling factor Brg1 onto target gene promoters compared to the wild-type Sp7. The mutant Sp7 also showed abrogation of the physical interaction of Sp7 with p300 and Brg1. Thus, p38-mediated phosphorylation of Sp7 may enhance the recruitment of its co-activators in the context of its “canonical” mode of action.

Another report showed that phosphorylation of Sp7 at Ser-73 and Ser-77 is necessary for its interaction with Fbw7 (F-box/WD repeat-containing protein 7), which works as a receptor subunit of the Skp1-Cullin1-F-box-protein (SCF)^Fbw7^ E3 ligase complex; Fbw7-targeted Sp7 undergoes ubiquitination and subsequent degradation [60]. Thus, p38-mediated phosphorylation directs Sp7 to degradation in this context.

Sp7 is acetylated at K307 and K312 by p300/CBP, and acetylation, in turn, enhances the stability of the Sp7 protein [61]. Sp7 acetylation is reversible; HDAC4 is likely to mediate deacetylation, as HDAC4 treatment accelerated Sp7 degradation. In EMSA, Sp7 binding to the GC-box was increased in the presence of CBP, whereas Sp7 mutants at acetylation sites showed a decreased binding property. Likewise, the Sp7 capacity for transactivation was enhanced in the presence of CBP, whereas this ability was attenuated in the Sp7 mutants. These data suggest that Sp7 acetylation at the two sites positively regulates the stability and transactivation ability of Sp7 in the context of its “canonical” mode of action. It remains to be examined whether the Sp7 phosphorylation and acetylation mentioned above affect its property in the “non-canonical” mode of action via AT-rich motifs.

Sirtuin 7 (Sirt7), one of the nicotinamide adenine dinucleotide (NAD)-dependent lysine deacylases, has been reported to regulate the transactivation property of Sp7 [62]. Global *Sirt7*-null mice showed decreased bone formation, and this phenotype was recapitulated by osteoblast-specific *Sirt7* deletion. Biochemical data suggest that Sirt7 increases the transcriptional activity of Sp7 in a DNA binding-independent manner through physical interaction. It is worth noting that Sirt7 did not affect the Sp7 function as a co-factor of Dlx5 and the Sp7 protein stability. From a mechanistic point of view, in vitro data suggest that Sirt7 causes deacylation of K368 in Sp7; the C-terminal deacylation is likely to enhance its N-terminal transactivation activity. K368 deacylation also facilitates Sirt1-mediated depropionylation of Sp7, which enhances its transactivation activity as well.

Sp7 has been shown to interact with other TFs in the osteoblast program. Physical interactions and synergistic functions between Sp7 and Runx2 have been demonstrated in the context of the transcription of osteoblast-related genes [43,63]. In addition, the enrichment of a Runx motif as well as the AT-rich motif in the Sp7 ChIP-seq [17] supports the Sp7–Runx2 interaction on the osteoblast genome.

p53 acts as a negative regulator of Sp7 in both its “canonical” and “non-canonical” modes of action [64]. Physical interaction of Sp7 with p53 inhibited not only Sp7 binding to the GC-box but also the Sp7–Dlx5 interaction. The interaction occurred between the p53 DNA-binding domain and part of the Sp7 transactivation domain proximal to its zinc fingers. Analysis using p53 mutants further demonstrated that the negative effect of p53 on Sp7 required a native conformation of p53, but not its DNA-binding ability. Given that p53 also represses Runx2 function [65], this study also suggests that the negative effect of p53 on osteoblastic gene expression depends on both Runx2 and Sp7.

A recent study highlighted zinc finger homeodomain 4 (Zfhx4) as a transcriptional partner of Sp7 at the late stage of endochondral ossification [66]. *Zfhx4*-null mice demonstrated impaired calcification of cartilage and reduced expression of the late hypertrophic chondrocyte marker Mmp13, which were similar to the cartilage phenotypes of *Sp7* mutant mice [43]. The interaction between Zfhx4 and Sp7 was then analyzed genetically and biochemically: heterozygous deletion of *Sp7* under the *Zfhx4*-null background led to a more severe impairment of chondrocyte maturation than was observed in the *Zfhx4*-null mice, and Zfhx4 physically interacted with Sp7 [66].

## 8. Relevance of SP7 to Human Skeletal Diseases

The significance of SP7 in osteogenesis has been confirmed in humans. Mutations in *SP7* have been identified as a rare cause of osteogenesis imperfecta (OI type XII) [67,68] in one case of juvenile Paget’s disease [69]. A genome-wide association study showed a significant association between variants in the *SP7* locus and bone mineral density [70]. The pathological mechanisms of *SP7*-mutation-caused diseases have been studied well in OI cases. OI comprises a group of connective tissue disorders characterized by bone fragility and low bone mass [67]. Although the majority (>90%) of patients with OI have autosomal dominant variants in *COL1A1* or *COL1A2*, numerous variants around other genes, including in *SP7*, have also been identified [67,68]. So far, three variants in *SP7* were identified to be associated with recessive OI cases. First, a homozygous single base pair deletion (p.Glu351GlyfsTer19) in *SP7* caused a frameshift, resulting in the removal of the last 81 amino acids of the protein in a patient with OI. The clinical features of this patient included recurrent fractures, mild bone deformities, delayed tooth eruption, and white sclera [71]. A second OI-associated homozygous mutation was identified in a patient who had p.Arg316Cys in *SP7*; the patient presented with osteoporosis, low-trauma fractures, and short stature [72]. The third mutation was p.Cys275Tyr in *SP7* [73]. In addition to these variants associated with recessive OI, a recent report showed a dominant form of OI caused by a heterozygous *SP7* variant (p.Glu340Ala) [74]. Low cortical density and cortical porosity were observed in these patients, consistent with previous reports of individuals with *SP7* mutations in recessive OI. However, the low bone turnover in the patients with dominant OI contrasts with the high turnover state seen in the previously reported patients with recessive OI. Recently, moreover, we and another group independently reported a dominant *SP7* mutation (p.Ser309Trp) in two individuals who showed sclerosis, bone fragility, and high turnover [69,75]. These cases presented with high bone mineral density and patchy sclerosis, which were different from the phenotypes in recessive OI. The impact of the variants on osteogenesis was confirmed by a mutant mouse line with the orthologous missense variant in *Sp7*. Its skeletal phenotype partially recapitulated the patient’s phenotypes [75].

Importantly, most of the variants described above were located in the zinc finger domain of *SP7*, and some were associated with the mode of action of SP7. Specifically, we found that the p.Ser309Trp variant increased the binding of SP7 to the GC-box and decreased the binding to an AT-rich motif [75]. A cross-comparison analysis of the activities of the *SP7* variants on the AT-rich motif through complex formation with DLX5 showed that SP7 carrying either the p.Ser309Trp or p.Glu340Ala variants decreased the activity on the AT-rich motif, compared to the wild type, whereas the p.Arg316Cys and p.Cys275Tyr variants did not significantly change the activity [74]. In considering all these results together, the fact that the different *SP7* variants were associated with different skeletal phenotypes indicates that the pathological phenotypes caused by *SP7* variants cannot be simply explained by the gain- or loss-of-function of Sp7. Rather, a complicated mode of SP7 action, possibly including both canonical and non-canonical modes, may underlie the pathological mechanism.

## 9. Future Perspectives

Mouse genetic studies and a large number of in vitro studies established Sp7 as an osteoblast determinant. As discussed in this review, additional layers of Sp7 actions have been unveiled over the past decade. Identification of the non-canonical mode of Sp7 action has given us a better understanding of the regulatory mechanism underlying Sp7-mediated osteoblast differentiation. It has also provided a new perspective on the pathological mechanisms underlying Sp7-associated bone diseases. However, the overall picture of Sp7-associated GRNs remains to be clarified. The next step could be defining Sp7 from the broader viewpoint of skeletal development and maintenance: How is Sp7 wired to skeletal GRNs depending on cell types and/or distinct phases of differentiation? How does Sp7 select one of the two modes of action in different situations? In the non-canonical mode of action, what are the Sp7 partners in the distinct cell types and differentiations? Recently advancing technologies for single-cell analyses and multiome analysis, including proteomics, will clarify these points, leading to the understanding of the pathological mechanisms underlying *SP7*-related skeletal diseases and the development of new therapeutic approaches for skeletal defects.

## Data Availability

Not applicable.

## References

[1] Moore K.L., Persaud T.V.N., Torchia M.G. (2020). The Developing Human: Clinically Oriented Embryology.

[2] Schoenwolf G.C., Bleyl S.B., Brauer P.R., Francis-West P.H., Larsen W.J. (2021). Larsen’s Human Embryology.

[3] Sadler T.W., Langman J. (2019). Langman’s Medical Embryology.

[4] Garzon-Alvarado D.A., Gutierrez M.L., Calixto L.F. (2014). A computational model of clavicle bone formation: A mechano-biochemical hypothesis. Bone.

[5] Long F., Ornitz D.M. (2013). Development of the endochondral skeleton. Cold Spring Harb. Perspect. Biol..

[6] Komori T., Yagi H., Nomura S., Yamaguchi A., Sasaki K., Deguchi K., Shimizu Y., Bronson R.T., Gao Y.H., Inada M. (1997). Targeted disruption of Cbfa1 results in a complete lack of bone formation owing to maturational arrest of osteoblasts. Cell.

[7] Nakashima K., Zhou X., Kunkel G., Zhang Z., Deng J.M., Behringer R.R., de Crombrugghe B. (2002). The novel zinc finger-containing transcription factor osterix is required for osteoblast differentiation and bone formation. Cell.

[8] Rodda S.J., McMahon A.P. (2006). Distinct roles for Hedgehog and canonical Wnt signaling in specification, differentiation and maintenance of osteoblast progenitors. Development.

[9] Maes C., Kobayashi T., Selig M.K., Torrekens S., Roth S.I., Mackem S., Carmeliet G., Kronenberg H.M. (2010). Osteoblast precursors, but not mature osteoblasts, move into developing and fractured bones along with invading blood vessels. Dev. Cell.

[10] Gao Y., Jheon A., Nourkeyhani H., Kobayashi H., Ganss B. (2004). Molecular cloning, structure, expression, and chromosomal localization of the human Osterix (SP7) gene. Gene.

[11] Nishio Y., Dong Y., Paris M., O’Keefe R.J., Schwarz E.M., Drissi H. (2006). Runx2-mediated regulation of the zinc finger Osterix/Sp7 gene. Gene.

[12] Nakashima K., de Crombrugghe B. (2003). Transcriptional mechanisms in osteoblast differentiation and bone formation. Trends Genet..

[13] Zhou X., Zhang Z., Feng J.Q., Dusevich V.M., Sinha K., Zhang H., Darnay B.G., de Crombrugghe B. (2010). Multiple functions of Osterix are required for bone growth and homeostasis in postnatal mice. Proc. Natl. Acad. Sci. USA.

[14] Baek W.Y., Lee M.A., Jung J.W., Kim S.Y., Akiyama H., de Crombrugghe B., Kim J.E. (2009). Positive regulation of adult bone formation by osteoblast-specific transcription factor osterix. J. Bone Miner. Res..

[15] Baek W.Y., de Crombrugghe B., Kim J.E. (2010). Postnatally induced inactivation of Osterix in osteoblasts results in the reduction of bone formation and maintenance. Bone.

[16] Yoshida C.A., Komori H., Maruyama Z., Miyazaki T., Kawasaki K., Furuichi T., Fukuyama R., Mori M., Yamana K., Nakamura K. (2012). SP7 inhibits osteoblast differentiation at a late stage in mice. PLoS ONE.

[17] Hojo H., Ohba S., He X., Lai L.P., McMahon A.P. (2016). Sp7/Osterix Is Restricted to Bone-Forming Vertebrates where It Acts as a Dlx Co-factor in Osteoblast Specification. Dev. Cell.

[18] Safe S., Abdelrahim M. (2005). Sp transcription factor family and its role in cancer. Eur. J. Cancer.

[19] Wang J., Zhuang J., Iyer S., Lin X., Whitfield T.W., Greven M.C., Pierce B.G., Dong X., Kundaje A., Cheng Y. (2012). Sequence features and chromatin structure around the genomic regions bound by 119 human transcription factors. Genome Res..

[20] Terrados G., Finkernagel F., Stielow B., Sadic D., Neubert J., Herdt O., Krause M., Scharfe M., Jarek M., Suske G. (2012). Genome-wide localization and expression profiling establish Sp2 as a sequence-specific transcription factor regulating vitally important genes. Nucleic Acids Res..

[21] Kennedy M.W., Chalamalasetty R.B., Thomas S., Garriock R.J., Jailwala P., Yamaguchi T.P. (2016). Sp5 and Sp8 recruit beta-catenin and Tcf1-Lef1 to select enhancers to activate Wnt target gene transcription. Proc. Natl. Acad. Sci. USA.

[22] Hume M.A., Barrera L.A., Gisselbrecht S.S., Bulyk M.L. (2015). UniPROBE, update 2015: New tools and content for the online database of protein-binding microarray data on protein-DNA interactions. Nucleic Acids Res..

[23] Wingender E., Schoeps T., Donitz J. (2013). TFClass: An expandable hierarchical classification of human transcription factors. Nucleic Acids Res..

[24] Hassan M.Q., Javed A., Morasso M.I., Karlin J., Montecino M., van Wijnen A.J., Stein G.S., Stein J.L., Lian J.B. (2004). Dlx3 transcriptional regulation of osteoblast differentiation: Temporal recruitment of Msx2, Dlx3, and Dlx5 homeodomain proteins to chromatin of the osteocalcin gene. Mol. Cell. Biol..

[25] Li H., Marijanovic I., Kronenberg M.S., Erceg I., Stover M.L., Velonis D., Mina M., Heinrich J.G., Harris S.E., Upholt W.B. (2008). Expression and function of Dlx genes in the osteoblast lineage. Dev. Biol..

[26] Tadic T., Dodig M., Erceg I., Marijanovic I., Mina M., Kalajzic Z., Velonis D., Kronenberg M.S., Kosher R.A., Ferrari D. (2002). Overexpression of Dlx5 in chicken calvarial cells accelerates osteoblastic differentiation. J. Bone Miner. Res..

[27] Perez-Gomez R., Fernández-Guerrero M., Campa V., Lopez-Gimenez J.F., Rada-Iglesias A., Ros M.A. (2020). Sp8 regulatory function in the limb bud ectoderm. bioRxiv.

[28] Rhodes C.S., Yoshitomi Y., Burbelo P.D., Freese N.H., Nakamura T., Chiba Y., Yamada Y., NIDCD/NIDCR Genomics and Computational Biology Core (2021). Sp6/Epiprofin is a master regulator in the developing tooth. Biochem. Biophys. Res. Commun..

[29] Ortuno M.J., Ruiz-Gaspa S., Rodriguez-Carballo E., Susperregui A.R., Bartrons R., Rosa J.L., Ventura F. (2010). p38 regulates expression of osteoblast-specific genes by phosphorylation of osterix. J. Biol. Chem..

[30] Koga T., Matsui Y., Asagiri M., Kodama T., de Crombrugghe B., Nakashima K., Takayanagi H. (2005). NFAT and Osterix cooperatively regulate bone formation. Nat. Med..

[31] Ortuno M.J., Susperregui A.R., Artigas N., Rosa J.L., Ventura F. (2013). Osterix induces Col1a1 gene expression through binding to Sp1 sites in the bone enhancer and proximal promoter regions. Bone.

[32] Yano H., Hamanaka R., Nakamura-Ota M., Adachi S., Zhang J.J., Matsuo N., Yoshioka H. (2014). Sp7/Osterix induces the mouse pro-alpha2(I) collagen gene (Col1a2) expression via the proximal promoter in osteoblastic cells. Biochem. Biophys. Res. Commun..

[33] Wu Y.F., Matsuo N., Sumiyoshi H., Yoshioka H. (2010). Sp7/Osterix is involved in the up-regulation of the mouse pro-alpha1(V) collagen gene (Col5a1) in osteoblastic cells. Matrix Biol..

[34] Yun-Feng W., Matsuo N., Sumiyoshi H., Yoshioka H. (2010). Sp7/Osterix up-regulates the mouse pro-alpha3(V) collagen gene (Col5a3) during the osteoblast differentiation. Biochem. Biophys. Res. Commun..

[35] Yang Y., Huang Y., Zhang L., Zhang C. (2016). Transcriptional regulation of bone sialoprotein gene expression by Osx. Biochem. Biophys. Res. Commun..

[36] Yang F., Tang W., So S., de Crombrugghe B., Zhang C. (2010). Sclerostin is a direct target of osteoblast-specific transcription factor osterix. Biochem. Biophys. Res. Commun..

[37] Niger C., Lima F., Yoo D.J., Gupta R.R., Buo A.M., Hebert C., Stains J.P. (2011). The transcriptional activity of osterix requires the recruitment of Sp1 to the osteocalcin proximal promoter. Bone.

[38] Onizuka S., Iwata T., Park S.J., Nakai K., Yamato M., Okano T., Izumi Y. (2016). ZBTB16 as a Downstream Target Gene of Osterix Regulates Osteoblastogenesis of Human Multipotent Mesenchymal Stromal Cells. J. Cell. Biochem..

[39] Han Y., Cho D.H., Chung D.J., Lee K.Y. (2016). Osterix plays a critical role in BMP4-induced promoter activity of connexin43. Biochem. Biophys. Res. Commun..

[40] Tang W., Yang F., Li Y., de Crombrugghe B., Jiao H., Xiao G., Zhang C. (2012). Transcriptional regulation of Vascular Endothelial Growth Factor (VEGF) by osteoblast-specific transcription factor Osterix (Osx) in osteoblasts. J. Biol. Chem..

[41] Yao B., Wang J., Qu S., Liu Y., Jin Y., Lu J., Bao Q., Li L., Yuan H., Ma C. (2019). Upregulated osterix promotes invasion and bone metastasis and predicts for a poor prognosis in breast cancer. Cell Death Dis..

[42] Zhang C., Tang W., Li Y. (2012). Matrix metalloproteinase 13 (MMP13) is a direct target of osteoblast-specific transcription factor osterix (Osx) in osteoblasts. PLoS ONE.

[43] Nishimura R., Wakabayashi M., Hata K., Matsubara T., Honma S., Wakisaka S., Kiyonari H., Shioi G., Yamaguchi A., Tsumaki N. (2012). Osterix regulates calcification and degradation of chondrogenic matrices through matrix metalloproteinase 13 (MMP13) expression in association with transcription factor Runx2 during endochondral ossification. J. Biol. Chem..

[44] Fu X., Li Y., Huang T., Yu Z., Ma K., Yang M., Liu Q., Pan H., Wang H., Wang J. (2018). Runx2/Osterix and Zinc Uptake Synergize to Orchestrate Osteogenic Differentiation and Citrate Containing Bone Apatite Formation. Adv. Sci..

[45] Lee Y.J., Park S.Y., Lee S.J., Boo Y.C., Choi J.Y., Kim J.E. (2015). Ucma, a direct transcriptional target of Runx2 and Osterix, promotes osteoblast differentiation and nodule formation. Osteoarthr. Cartil..

[46] Gao M.M., Su Q.N., Liang T.Z., Ma J.X., Liang T.Z., Stoddart M.J., Richards R.G., Zhou Z.Y., Zou N.X. (2018). Transcriptional activation of ENPP1 by osterix in osteoblasts and osteocytes. Eur. Cell Mater..

[47] Liu Q., Li M., Wang S., Xiao Z., Xiong Y., Wang G. (2020). Recent Advances of Osterix Transcription Factor in Osteoblast Differentiation and Bone Formation. Front. Cell Dev. Biol..

[48] Kawane T., Komori H., Liu W., Moriishi T., Miyazaki T., Mori M., Matsuo Y., Takada Y., Izumi S., Jiang Q. (2014). Dlx5 and mef2 regulate a novel runx2 enhancer for osteoblast-specific expression. J. Bone Miner. Res..

[49] Bedalov A., Salvatori R., Dodig M., Kronenberg M.S., Kapural B., Bogdanovic Z., Kream B.E., Woody C.O., Clark S.H., Mack K. (1995). Regulation of COL1A1 expression in type I collagen producing tissues: Identification of a 49 base pair region which is required for transgene expression in bone of transgenic mice. J. Bone Miner. Res..

[50] Oh J.H., Park S.Y., de Crombrugghe B., Kim J.E. (2012). Chondrocyte-specific ablation of Osterix leads to impaired endochondral ossification. Biochem. Biophys. Res. Commun..

[51] Xing W., Godwin C., Pourteymoor S., Mohan S. (2019). Conditional disruption of the osterix gene in chondrocytes during early postnatal growth impairs secondary ossification in the mouse tibial epiphysis. Bone Res..

[52] Moriishi T., Ito T., Fukuyama R., Qin X., Komori H., Kaneko H., Matsuo Y., Yoshida N., Komori T. (2022). Sp7 Transgenic Mice with a Markedly Impaired Lacunocanalicular Network Induced Sost and Reduced Bone Mass by Unloading. Int. J. Mol. Sci..

[53] Wang J.S., Kamath T., Mazur C.M., Mirzamohammadi F., Rotter D., Hojo H., Castro C.D., Tokavanich N., Patel R., Govea N. (2021). Control of osteocyte dendrite formation by Sp7 and its target gene osteocrin. Nat. Commun..

[54] Buenzli P.R., Sims N.A. (2015). Quantifying the osteocyte network in the human skeleton. Bone.

[55] Matsubara T., Kida K., Yamaguchi A., Hata K., Ichida F., Meguro H., Aburatani H., Nishimura R., Yoneda T. (2008). BMP2 regulates Osterix through Msx2 and Runx2 during osteoblast differentiation. J. Biol. Chem..

[56] Salazar V.S., Capelo L.P., Cantu C., Zimmerli D., Gosalia N., Pregizer S., Cox K., Ohte S., Feigenson M., Gamer L. (2019). Reactivation of a developmental Bmp2 signaling center is required for therapeutic control of the murine periosteal niche. Elife.

[57] Hojo H., Saito T., He X., Guo Q., Onodera S., Azuma T., Koebis M., Nakao K., Aiba A., Seki M. (2021). Runx2 Regulates Chromatin Accessibility to Direct Skeletal Cell Programs. Sneak Peek.

[58] He S., Yang S., Zhang Y., Li X., Gao D., Zhong Y., Cao L., Ma H., Liu Y., Li G. (2019). LncRNA ODIR1 inhibits osteogenic differentiation of hUC-MSCs through the FBXO25/H2BK120ub/H3K4me3/OSX axis. Cell Death Dis..

[59] Sun Y., Cai M., Zhong J., Yang L., Xiao J., Jin F., Xue H., Liu X., Liu H., Zhang Y. (2019). The long noncoding RNA lnc-ob1 facilitates bone formation by upregulating Osterix in osteoblasts. Nat. Metab..

[60] Hoshikawa S., Shimizu K., Watahiki A., Chiba M., Saito K., Wei W., Fukumoto S., Inuzuka H. (2020). Phosphorylation-dependent osterix degradation negatively regulates osteoblast differentiation. FASEB J..

[61] Lu J., Qu S., Yao B., Xu Y., Jin Y., Shi K., Shui Y., Pan S., Chen L., Ma C. (2016). Osterix acetylation at K307 and K312 enhances its transcriptional activity and is required for osteoblast differentiation. Oncotarget.

[62] Fukuda M., Yoshizawa T., Karim M.F., Sobuz S.U., Korogi W., Kobayasi D., Okanishi H., Tasaki M., Ono K., Sawa T. (2018). SIRT7 has a critical role in bone formation by regulating lysine acylation of SP7/Osterix. Nat. Commun..

[63] Rashid H., Ma C., Chen H., Wang H., Hassan M.Q., Sinha K., de Crombrugghe B., Javed A. (2014). Sp7 and Runx2 molecular complex synergistically regulate expression of target genes. Connect. Tissue Res..

[64] Artigas N., Gamez B., Cubillos-Rojas M., Sanchez-de Diego C., Valer J.A., Pons G., Rosa J.L., Ventura F. (2017). p53 inhibits SP7/Osterix activity in the transcriptional program of osteoblast differentiation. Cell Death Differ..

[65] Van der Deen M., Taipaleenmaki H., Zhang Y., Teplyuk N.M., Gupta A., Cinghu S., Shogren K., Maran A., Yaszemski M.J., Ling L. (2013). MicroRNA-34c inversely couples the biological functions of the runt-related transcription factor RUNX2 and the tumor suppressor p53 in osteosarcoma. J. Biol. Chem..

[66] Nakamura E., Hata K., Takahata Y., Kurosaka H., Abe M., Abe T., Kihara M., Komori T., Kobayashi S., Murakami T. (2021). Zfhx4 regulates endochondral ossification as the transcriptional platform of Osterix in mice. Commun. Biol..

[67] Marini J.C., Forlino A., Bachinger H.P., Bishop N.J., Byers P.H., Paepe A., Fassier F., Fratzl-Zelman N., Kozloff K.M., Krakow D. (2017). Osteogenesis imperfecta. Nat. Rev. Dis. Primers.

[68] Marini J.C., Dang Do A.N. (2020). Osteogenesis Imperfecta.

[69] Whyte M.P., Campeau P.M., McAlister W.H., Roodman G.D., Kurihara N., Nenninger A., Duan S., Gottesman G.S., Bijanki V.N., Sedighi H. (2020). Juvenile Paget’s Disease From Heterozygous Mutation of SP7 Encoding Osterix (Specificity Protein 7, Transcription Factor SP7). Bone.

[70] Timpson N.J., Tobias J.H., Richards J.B., Soranzo N., Duncan E.L., Sims A.M., Whittaker P., Kumanduri V., Zhai G., Glaser B. (2009). Common variants in the region around Osterix are associated with bone mineral density and growth in childhood. Hum. Mol. Genet..

[71] Lapunzina P., Aglan M., Temtamy S., Caparros-Martin J.A., Valencia M., Leton R., Martinez-Glez V., Elhossini R., Amr K., Vilaboa N. (2010). Identification of a frameshift mutation in Osterix in a patient with recessive osteogenesis imperfecta. Am. J. Hum. Genet..

[72] Fiscaletti M., Biggin A., Bennetts B., Wong K., Briody J., Pacey V., Birman C., Munns C.F. (2018). Novel variant in Sp7/Osx associated with recessive osteogenesis imperfecta with bone fragility and hearing impairment. Bone.

[73] Hayat A., Hussain S., Bilal M., Kausar M., Almuzzaini B., Abbas S., Tanveer A., Khan A., Siddiqi S., Foo J.N. (2020). Biallelic variants in four genes underlying recessive osteogenesis imperfecta. Eur. J. Med. Genet..

[74] Ludwig K., Ward L.M., Khan N., Robinson M.E., Miranda V., Bardai G., Moffatt P., Rauch F. (2022). Dominant osteogenesis imperfecta with low bone turnover caused by a heterozygous SP7 variant. Bone.

[75] Lui J.C., Raimann A., Hojo H., Dong L., Roschger P., Kikani B., Wintergerst U., Fratzl-Zelman N., Jee Y.H., Haeusler G. (2022). A neomorphic variant in SP7 alters sequence specificity and causes a high-turnover bone disorder. Nat. Commun..

